# Supplementary material for: Iterative improvement in the automatic modular design of robot swarms
Source: PeerJ Comput Sci. 2020 Dec 7;6:e322. doi: 10.7717/peerj-cs.322 (PMC7924708; doi:10.7717/peerj-cs.322)
Supplement: Supplemental Information 3 [file peerj-cs-06-322-s003.zip › argos3/doc/api/standalone/a00629_source.html]

ARGoS: plugins/simulator/visualizations/qt-opengl/models/qtopengl\_box.cpp Source File


- Main Page
- Related Pages
- Namespaces
- Classes
- Files

- File List
- File Members

# plugins/simulator/visualizations/qt-opengl/models/qtopengl\_box.cpp

Go to the documentation of this file.

```
00001 
00007 #include "qtopengl_box.h"
00008 #include <argos3/core/utility/math/vector2.h>
00009 #include <argos3/plugins/simulator/entities/led_equipped_entity.h>
00010 #include <argos3/plugins/simulator/entities/box_entity.h>
00011 #include <argos3/plugins/simulator/visualizations/qt-opengl/qtopengl_widget.h>
00012 
00013 namespace argos {
00014 
00015    /****************************************/
00016    /****************************************/
00017 
00018    static const Real LED_RADIUS     = 0.01f;
00019    const GLfloat MOVABLE_COLOR[]    = { 1.0f, 0.0f, 0.0f, 1.0f };
00020    const GLfloat NONMOVABLE_COLOR[] = { 0.7f, 0.7f, 0.7f, 1.0f };
00021    const GLfloat SPECULAR[]         = { 0.0f, 0.0f, 0.0f, 1.0f };
00022    const GLfloat SHININESS[]        = { 0.0f                   };
00023    const GLfloat EMISSION[]         = { 0.0f, 0.0f, 0.0f, 1.0f };
00024 
00025    /****************************************/
00026    /****************************************/
00027 
00028     CQTOpenGLBox::CQTOpenGLBox() :
00029        m_unVertices(20){
00030 
00031        /* Reserve the needed display lists */
00032        m_unBaseList = glGenLists(2);
00033        m_unBodyList = m_unBaseList;
00034        m_unLEDList = m_unBaseList + 1;
00035 
00036        /* Make body list */
00037        glNewList(m_unBodyList, GL_COMPILE);
00038        MakeBody();
00039        glEndList();
00040 
00041        /* Make LED list */
00042        glNewList(m_unLEDList, GL_COMPILE);
00043        MakeLED();
00044        glEndList();
00045 
00046     }
00047 
00048    /****************************************/
00049    /****************************************/
00050 
00051    CQTOpenGLBox::~CQTOpenGLBox() {
00052       glDeleteLists(m_unBaseList, 2);
00053    }
00054 
00055    /****************************************/
00056    /****************************************/
00057 
00058    void CQTOpenGLBox::DrawLEDs(CBoxEntity& c_entity) {
00059       /* Draw the LEDs */
00060       GLfloat pfColor[]           = {   0.0f, 0.0f, 0.0f, 1.0f };
00061       const GLfloat pfSpecular[]  = {   0.0f, 0.0f, 0.0f, 1.0f };
00062       const GLfloat pfShininess[] = { 100.0f                   };
00063       const GLfloat pfEmission[]  = {   0.0f, 0.0f, 0.0f, 1.0f };
00064       glMaterialfv(GL_FRONT_AND_BACK, GL_SPECULAR, pfSpecular);
00065       glMaterialfv(GL_FRONT_AND_BACK, GL_SHININESS, pfShininess);
00066       glMaterialfv(GL_FRONT_AND_BACK, GL_EMISSION, pfEmission);
00067       CLEDEquippedEntity& cLEDEquippedEntity = c_entity.GetLEDEquippedEntity();
00068       for(UInt32 i = 0; i < cLEDEquippedEntity.GetLEDs().size(); ++i) {
00069          glPushMatrix();
00070          /* Set the material */
00071          const CColor& cColor = cLEDEquippedEntity.GetLED(i).GetColor();
00072          pfColor[0] = cColor.GetRed()   / 255.0f;
00073          pfColor[1] = cColor.GetGreen() / 255.0f;
00074          pfColor[2] = cColor.GetBlue()  / 255.0f;
00075          glMaterialfv(GL_FRONT_AND_BACK, GL_AMBIENT_AND_DIFFUSE, pfColor);
00076          /* Perform rototranslation */
00077          const CVector3& cPosition = cLEDEquippedEntity.GetLEDOffset(i);
00078          glTranslatef(cPosition.GetX(), cPosition.GetY(), cPosition.GetZ());
00079          /* Draw the LED */
00080          glCallList(m_unLEDList);
00081          glPopMatrix();
00082       }
00083    }
00084 
00085    /****************************************/
00086    /****************************************/
00087 
00088    void CQTOpenGLBox::Draw(const CBoxEntity& c_entity) {
00089       /* Draw the body */
00090       if(c_entity.GetEmbodiedEntity().IsMovable()) {
00091          glMaterialfv(GL_FRONT_AND_BACK, GL_AMBIENT_AND_DIFFUSE, MOVABLE_COLOR);
00092       }
00093       else {
00094          glMaterialfv(GL_FRONT_AND_BACK, GL_AMBIENT_AND_DIFFUSE, NONMOVABLE_COLOR);
00095       }
00096       glPushMatrix();
00097       glScalef(c_entity.GetSize().GetX(), c_entity.GetSize().GetY(), c_entity.GetSize().GetZ());
00098       glCallList(m_unBodyList);
00099       glPopMatrix();
00100    }
00101 
00102    /****************************************/
00103    /****************************************/
00104 
00105    void CQTOpenGLBox::MakeBody() {
00106              /* Since this shape can be stretched,
00107                  make sure the normal vectors are unit-long */
00108               glEnable(GL_NORMALIZE);
00109 
00110               /* Set the material */
00111               glMaterialfv(GL_FRONT_AND_BACK, GL_SPECULAR, SPECULAR);
00112               glMaterialfv(GL_FRONT_AND_BACK, GL_SHININESS, SHININESS);
00113               glMaterialfv(GL_FRONT_AND_BACK, GL_EMISSION, EMISSION);
00114 
00115               /* Let's start the actual shape */
00116 
00117               /* This part covers the top and bottom faces (parallel to XY) */
00118               glBegin(GL_QUADS);
00119               /* Bottom face */
00120               glNormal3f(0.0f, 0.0f, -1.0f);
00121               glVertex3f( 0.5f,  0.5f, 0.0f);
00122               glVertex3f( 0.5f, -0.5f, 0.0f);
00123               glVertex3f(-0.5f, -0.5f, 0.0f);
00124               glVertex3f(-0.5f,  0.5f, 0.0f);
00125               /* Top face */
00126               glNormal3f(0.0f, 0.0f, 1.0f);
00127               glVertex3f(-0.5f, -0.5f, 1.0f);
00128               glVertex3f( 0.5f, -0.5f, 1.0f);
00129               glVertex3f( 0.5f,  0.5f, 1.0f);
00130               glVertex3f(-0.5f,  0.5f, 1.0f);
00131               glEnd();
00132               /* This part covers the faces (South, East, North, West) */
00133               glBegin(GL_QUADS);
00134               /* South face */
00135         glNormal3f(0.0f, -1.0f, 0.0f);
00136         glVertex3f(-0.5f, -0.5f, 1.0f);
00137         glVertex3f(-0.5f, -0.5f, 0.0f);
00138         glVertex3f( 0.5f, -0.5f, 0.0f);
00139         glVertex3f( 0.5f, -0.5f, 1.0f);
00140         /* East face */
00141         glNormal3f(1.0f, 0.0f, 0.0f);
00142         glVertex3f( 0.5f, -0.5f, 1.0f);
00143         glVertex3f( 0.5f, -0.5f, 0.0f);
00144         glVertex3f( 0.5f,  0.5f, 0.0f);
00145         glVertex3f( 0.5f,  0.5f, 1.0f);
00146         /* North face */
00147         glNormal3f(0.0f, 1.0f, 0.0f);
00148         glVertex3f( 0.5f,  0.5f, 1.0f);
00149         glVertex3f( 0.5f,  0.5f, 0.0f);
00150         glVertex3f(-0.5f,  0.5f, 0.0f);
00151         glVertex3f(-0.5f,  0.5f, 1.0f);
00152         /* West face */
00153         glNormal3f(-1.0f, 0.0f, 0.0f);
00154         glVertex3f(-0.5f,  0.5f, 1.0f);
00155         glVertex3f(-0.5f,  0.5f, 0.0f);
00156         glVertex3f(-0.5f, -0.5f, 0.0f);
00157         glVertex3f(-0.5f, -0.5f, 1.0f);
00158               glEnd();
00159               /* The shape definitions is finished */
00160 
00161               /* We don't need it anymore */
00162               glDisable(GL_NORMALIZE);
00163    }
00164 
00165    /****************************************/
00166    /****************************************/
00167 
00168    void CQTOpenGLBox::MakeLED() {
00169       CVector3 cNormal, cPoint;
00170       CRadians cSlice(CRadians::TWO_PI / m_unVertices);
00171 
00172       glBegin(GL_TRIANGLE_STRIP);
00173       for(CRadians cInclination; cInclination <= CRadians::PI; cInclination += cSlice) {
00174          for(CRadians cAzimuth; cAzimuth <= CRadians::TWO_PI; cAzimuth += cSlice) {
00175 
00176             cNormal.FromSphericalCoords(1.0f, cInclination, cAzimuth);
00177             cPoint = LED_RADIUS * cNormal;
00178             glNormal3f(cNormal.GetX(), cNormal.GetY(), cNormal.GetZ());
00179             glVertex3f(cPoint.GetX(), cPoint.GetY(), cPoint.GetZ());
00180 
00181             cNormal.FromSphericalCoords(1.0f, cInclination + cSlice, cAzimuth);
00182             cPoint = LED_RADIUS * cNormal;
00183             glNormal3f(cNormal.GetX(), cNormal.GetY(), cNormal.GetZ());
00184             glVertex3f(cPoint.GetX(), cPoint.GetY(), cPoint.GetZ());
00185 
00186             cNormal.FromSphericalCoords(1.0f, cInclination, cAzimuth + cSlice);
00187             cPoint = LED_RADIUS * cNormal;
00188             glNormal3f(cNormal.GetX(), cNormal.GetY(), cNormal.GetZ());
00189             glVertex3f(cPoint.GetX(), cPoint.GetY(), cPoint.GetZ());
00190 
00191             cNormal.FromSphericalCoords(1.0f, cInclination + cSlice, cAzimuth + cSlice);
00192             cPoint = LED_RADIUS * cNormal;
00193             glNormal3f(cNormal.GetX(), cNormal.GetY(), cNormal.GetZ());
00194             glVertex3f(cPoint.GetX(), cPoint.GetY(), cPoint.GetZ());
00195 
00196          }
00197       }
00198       glEnd();
00199    }
00200 
00201    /****************************************/
00202    /****************************************/
00203 
00204    class CQTOpenGLOperationDrawBoxNormal : public CQTOpenGLOperationDrawNormal {
00205    public:
00206       void ApplyTo(CQTOpenGLWidget& c_visualization,
00207                    CBoxEntity& c_entity) {
00208          static CQTOpenGLBox m_cModel;
00209          c_visualization.DrawEntity(c_entity.GetEmbodiedEntity());
00210          m_cModel.Draw(c_entity);
00211          m_cModel.DrawLEDs(c_entity);
00212       }
00213    };
00214 
00215    class CQTOpenGLOperationDrawBoxSelected : public CQTOpenGLOperationDrawSelected {
00216    public:
00217       void ApplyTo(CQTOpenGLWidget& c_visualization,
00218                    CBoxEntity& c_entity) {
00219          c_visualization.DrawBoundingBox(c_entity.GetEmbodiedEntity());
00220       }
00221    };
00222 
00223    REGISTER_QTOPENGL_ENTITY_OPERATION(CQTOpenGLOperationDrawNormal, CQTOpenGLOperationDrawBoxNormal, CBoxEntity);
00224 
00225    REGISTER_QTOPENGL_ENTITY_OPERATION(CQTOpenGLOperationDrawSelected, CQTOpenGLOperationDrawBoxSelected, CBoxEntity);
00226 
00227    /****************************************/
00228    /****************************************/
00229 
00230 }
```

---

Generated on 10 Jul 2018 for ARGoS by 
 1.6.1
